# A Squirrel’s Guide to the Olive Galaxy: Tree-Level Determinants of Den-Site Selection in the Persian Squirrel within Traditional Mediterranean Olive Groves

**DOI:** 10.3390/biology14121676

**Published:** 2025-11-25

**Authors:** Yiannis G. Zevgolis, Efstratios Kamatsos, Apostolos Christopoulos, Christina Valeta, Eleni Rekouti, Christos Xagoraris, George P. Mitsainas, Petros Lymberakis, Dionisios Youlatos, Panayiotis G. Dimitrakopoulos

**Affiliations:** 1 Biodiversity Conservation Laboratory, Department of Environment, University of the Aegean, GR-81132 Mytilene, Greece; kamatsos.stratos@gmail.com (E.K.); xristinavaleta@gmail.com (C.V.); pdimi@aegean.gr (P.G.D.); 2 Department of Zoology and Marine Biology, Faculty of Biology, National and Kapodistrian University of Athens, GR-15772 Athens, Greece; apochris@biol.uoa.gr; 3 Section of Animal Biology, Department of Biology, University of Patras, GR-26504 Patras, Greece; elrekouti@gmail.com (E.R.); mitsain@upatras.gr (G.P.M.); 4 Department of Geography, School of Environment, Geography and Applied Economics, Harokopio University, GR-17671 Athens, Greece; cxago@hua.gr; 5 Natural History Museum of Crete, School of Sciences and Engineering, University of Crete, GR-71409 Irakleio, Greece; lyberis@nhmc.uoc.gr; 6 Laboratory of Marine and Terrestrial Animal Diversity, Department of Zoology, School of Biology, Aristotle University of Thessaloniki, GR-54124 Thessaloniki, Greece; dyoul@bio.auth.gr; 7 International Center for Biodiversity and Primate Conservation, Dali University, Dali 671003, China

**Keywords:** arboreal mammal ecology, centennial olive trees, den-site suitability, infrared thermography, insular Mediterranean ecosystems, microclimatic buffering, *Sciurus anomalus*, tree functional traits, High Nature Value farmlands

## Abstract

**Simple Summary:**

Traditional centennial olive groves represent ecologically complex agroecosystems that combine agricultural use with high conservation value across the Mediterranean Basin. On Lesvos Island, Greece, which is the westernmost limit of the Persian squirrel’s (*Sciurus anomalus*) natural range, these centennial olive trees provide essential nesting refuges. To identify the factors guiding den-site selection, we measured structural, physiological, and thermal traits of 288 olive trees, including 36 confirmed den trees. Squirrels consistently selected older and taller trees with extensive crowns, high leaf area, and elevated photosynthetic performance, traits that enhance canopy productivity and promote a more stable microclimate. Infrared thermography further revealed that den trees exhibited lower orientation-related temperature asymmetries, reflecting stronger thermal regulation at the trunk level. Integrative modeling identified crown development and trunk thermal stability as the most decisive predictors of den occupancy. These results demonstrate that centennial olive trees function as important biophysical refuges, reinforcing the need to preserve traditional olive landscapes as key habitats for arboreal mammals in Mediterranean ecosystems.

**Abstract:**

Traditional centennial olive groves represent ecologically valuable agroecosystems that support both biodiversity and cultural heritage across Mediterranean landscapes. On Lesvos Island, Greece, which marks the westernmost limit of the Persian squirrel (*Sciurus anomalus*) distribution, these centennial olive trees serve as essential nesting resources for this regionally Vulnerable species. However, the tree-level mechanisms determining den-site suitability remain insufficiently understood. We examined 288 centennial olive trees, including 36 with confirmed dens, integrating structural, physiological, and thermal metrics to identify the attributes influencing den occupancy. Our results showed that squirrels consistently selected older and taller olives with broad crowns and high photosynthetic activity, indicating a preference for vigorous, architecturally complex trees that provide stable microclimatic conditions. Infrared thermography revealed that occupied trees exhibited lower trunk temperature asymmetries and stronger thermal buffering capacity, highlighting the role of microclimatic stability in den-site selection. Overall, our findings show that den-site selection in *S. anomalus* is shaped by the interplay of structural maturity, physiological performance, and thermal coherence. By linking tree function to den-site suitability, our work advances a mechanistic understanding of microhabitat selection and emphasizes the importance of centennial olive trees as biophysical refugia within traditional Mediterranean agroecosystems.

## 1. Introduction

The transformation of natural landscapes into agricultural ones through centuries of human–environment interaction has given rise to traditional agroecosystems. These are spatially and temporally complex systems that reflect a long-term coadaptation between ecological processes and human land use [1,2]. In the face of accelerating global biodiversity loss, these agroecosystems, now formally recognized under the European framework of High Nature Value farmlands (HNVfs) [3,4], are increasingly regarded not merely as remnants of traditional agriculture, but as multifunctional landscapes that integrate agricultural productivity with high habitat heterogeneity [2,5]. As such, they maintain higher biodiversity than intensive agricultural systems and exhibit considerable adaptive capacity under changing climatic conditions [6,7,8]. This inherent complexity enables them to sustain structurally diverse habitats, support rich faunal assemblages across taxonomic groups, and maintain essential ecological functions across various spatial scales. Therefore, they are increasingly recognized as critical refugia for species persistence in human-modified environments [4,9], underscoring their growing importance in conservation science [10,11].

Among the most paradigmatic forms of HNVf in the Mediterranean Basin, traditional olive groves stand out as ecologically valuable agroecosystems, shaped by centuries of low-intensity cropping methods [9,12,13,14], and adapted over time to shifting agricultural practices and environmental conditions [15,16,17]. These structurally complex systems perform a variety of ecosystem services, including the conservation of soil quality [18,19], mitigation of erosion [20], enhancement of local water retention [21], and carbon sequestration [22,23]. They often comprise widely spaced trees [24] interspersed with semi-natural vegetation, and traditional landscape infrastructures such as terraces and dry-stone walls [25,26]; thus, despite pressures from land abandonment [8,27,28,29,30,31,32], modernization [17,33,34], and intensification of cultivation practices [35,36], traditional olive groves have demonstrated remarkable ecological resilience [12,37], continuing to function as multifunctional landscapes of high conservation value.

At the core of these multifunctional groves, centennial olive trees act as keystone elements, contributing not only to landscape heterogeneity but also providing distinctive structural complexity, long-term physiological resilience and prolonged functional lifespan [32,38], which together generate a mosaic of microhabitat conditions essential for the persistence of a wide range of organisms [39,40,41,42]. The ecological value of these trees, however, is most clearly expressed through their architectural attributes, which accumulate over centuries of growth and interaction with natural and anthropogenic processes. Unlike younger or intensively pruned individuals, these senescent trees typically exhibit pronounced architectural diversity, including irregular branching patterns, canopy asymmetry, gnarled trunks, deep bark fissures, large scaffolding branches, hollow sections, and decaying wood [14,38]. These features arise from long-term internal compartmentalization processes [43,44] in response to mechanical injury, pathogenic attack, or abiotic stress, and result in the formation of internal cavities and refugia [45], and are increasingly recognized not as signs of decline but as indicators of ecological maturity [46].

These structural transformations not only signal the ecological maturity of centennial olive trees but also actively shape the conditions within and around them. By modifying light diffusion, thermal exposure, and humidity dynamics within and around the tree crown [47,48], and in combination with traditional management practices such as rotational pruning and controlled grazing, which together foster physiological longevity and habitat heterogeneity [12,26], they form complex microhabitats essential for cavity-nesting vertebrates, saproxylic insects, epiphytes, and microbial communities [39]. Consequently, centennial olive trees function as ecological analogues of veteran forest trees, providing persistent nesting substrates, thermally buffered microclimates, and biodiversity-supporting cavities, particularly in rural Mediterranean landscapes where natural woodland elements have diminished [9,32].

Collectively, these attributes establish centennial olive trees as unique microclimatic refuges and secure nesting substrates for species with specialized microhabitat requirements. Yet, the very processes that generate cavities and decaying wood also pose challenges to tree health [47,49,50] by disrupting vascular integrity and resource allocation [48,51,52] and thereby diminishing productivity and physiological function over time [53,54,55]. Nonetheless, these structural anomalies dramatically increase diversity of microhabitats at the tree level, creating a mosaic of ecological niches that support diverse taxa [13,36,46] and ultimately reinforcing the pivotal role of centennial olive trees as biodiversity-supporting keystone structures within anthropogenic landscapes [14,39].

Among the many species that rely on such structural anomalies of centennial olive trees [56,57], small mammals, including cavity-dependent arboreal rodents, exhibit particularly strong ecological associations with these centennial trees. One such species is the Persian squirrel (*Sciurus anomalus*), a southwest Asian arboreal mammal whose range extends from the eastern Mediterranean to the Middle East, encompassing Turkey (including the Aegean island of Gökçeada), the Caucasus, Iran, and Jordan [58,59,60]. Greece hosts the westernmost population of the species, which is confined to the island of Lesvos and represents its only European occurrence [61]. Though globally classified as Least Concern, *S. anomalus* faces considerable regional pressures, such as agricultural intensification, road development, wildfires, and predation by feral cats [60,62,63,64], many of which are exacerbated at the periphery of its distribution [41,60], where population declines have already been documented; consequently, the Greek population has been reclassified as Vulnerable [65]. In such marginal, fragmented habitats, the availability and quality of nesting resources, particularly tree hollows and cavities, may critically influence population viability, as these features offer essential protection from predators, thermal extremes, and human disturbance.

In these contexts, access to suitable nesting substrates becomes a limiting factor for arboreal squirrels, influencing their spatial distribution, reproductive success, and long-term persistence. For *S. anomalus*, cavity-bearing trees, and in particular oaks, olives, and other broadleaved species, constitute key nesting resources across much of its range [61,62,66,67]. These arboreal refuges serve multiple vital functions: shelter from thermal extremes and predators, sites for resting and breeding, and secure spaces for raising young [68,69,70,71]. In the Mediterranean, where climatic stressors, such as heat and drought prevail, tree cavities offer microclimatic buffering and security far superior to exposed branch nests. For *S. anomalus*, such cavities are typically located at heights of 5–14 m, with entrance holes oriented toward the south or southeast to optimize thermal regulation [72,73], and with narrow external openings that reduce predation risk (Y.G.Z., pers. obs.). Although individuals may shift nests seasonally, likely to reduce ectoparasite loads, they frequently reuse the same cavities across years [73]. Thus, den-site availability, architecture, and positioning are not incidental but represent integral components of the species’ behavioral ecology and survival strategies.

Yet, despite this recognized importance, the den-site ecology of *S. anomalus* within traditional olive groves remains poorly understood. In particular, although centennial olive trees dominate many Mediterranean rural landscapes, their role in the denning ecology of *S. anomalus* has never been empirically assessed. While prior research has addressed broader habitat associations and species distribution at landscape scales [41,74], the fine-scale decision-making processes governing den-site selection remain unexplored. Moreover, despite the increasing recognition of microclimatic conditions as key determinants of den-site quality in arboreal mammals, no study to date has evaluated how surface temperature regimes differ between occupied and unoccupied trees in HNV traditional olive groves.

To address these gaps, we conducted a comparative, tree-level study in traditional centennial olive groves on the island of Lesvos, Greece, explicitly contrasting occupied (“den”) trees with neighboring unoccupied trees through non-destructive phenotyping and infrared thermography (IRT). Our objectives were fourfold: (1) to identify which structural and physiological traits of centennial olive trees are most strongly associated with den occupancy by *S. anomalus*; (2) to evaluate the role of tree-level thermal microclimates, derived from IRT of trunks and crowns, in explaining patterns of den occupancy; (3) to characterize the overall multivariate separation between occupied and unoccupied trees; and (4) to develop an integrated predictive framework that combines structural physiological, and thermal attributes to assess den-site suitability at the tree level for *S. anomalus* within traditional olive landscapes.

## 2. Materials and Methods

### 2.1. Study Area

**Figure 1:**
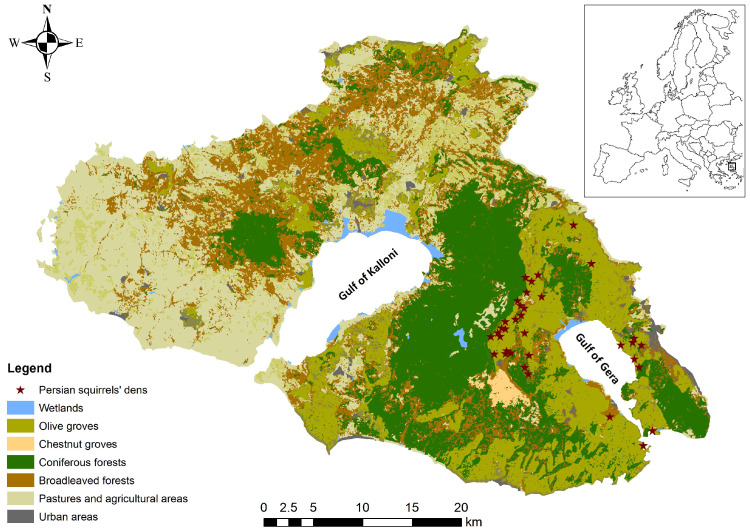
Map of the island of Lesvos (NE Aegean, Greece) showing major habitat types and the confirmed den sites (red stars) of the Persian squirrel (*Sciurus anomalus*) within High Nature Value (HNV) olive areas. Inset: geographic location of Lesvos within Europe.

Lesvos Island, situated in the northeastern Aegean Sea less than 10 km from the Anatolian mainland, is the third largest island of Greece (1632.8 km^2^) and the eighth largest in the Mediterranean. Its semi-mountainous terrain peaks at Mt. Lepetymnos (968 m) in the north and Mt. Olympus (967 m) in the center, while its heterogeneous landscapes encompass extensive Calabrian pine (*Pinus brutia*) and European black pine (*P. nigra*) forests, scattered oak woodlands (*Quercus ithaburensis*, *Q. coccifera*), Mediterranean maquis and phrygana, wetlands [75,76,77], and expansive traditional olive groves (*Olea europaea*) (Figure 1). This biogeographic setting at the interface of the Mediterranean and Anatolian regions fosters unusually high species richness, combining taxa of both origins [64].

The island’s climate is Mediterranean, with cool, wet winters and hot, dry summers. Mean monthly temperatures range from 9.6 °C in January to 27.0 °C in July, and annual precipitation averages 645 mm, concentrated between October and April [78]. This seasonality imposes significant ecological pressures on arboreal mammals, as prolonged summer drought and elevated ambient temperatures intensify physiological stress and predation risk, thereby amplifying the functional importance of microclimatic refugia, such as tree cavities.

Within this climatic context, olive cultivation emerges as the dominant land use shaping both habitat availability and the distribution of such refugia, as it dominates the agricultural landscape of Lesvos, covering 415.7 km^2^ (≈25% of the island’s area and ≈87% of its agricultural land) [55] with an estimated 8–11 million trees [16]. Within the island, centennial olive groves represent structurally mature, low-intensity agroecosystems shaped by centuries of traditional management, including terracing and dry-stone walling, without irrigation, chemical inputs, or mechanized interventions [13]. These groves not only constitute one of the largest continuous HNVf agroecosystems in the Aegean but also provide the most extensive source of cavity-bearing trees across the island, as broadleaved forests are patchy and limited in spatial extent compared to the widespread distribution of olive groves. Unlike intensively managed groves, these traditional systems maintain a long-term continuity of individual trees, most of which are several centuries old and persist through repeated cycles of resprouting from the same ancient rootstocks. Although chronologically similar, these trees exhibit pronounced divergence in their structural and physiological trajectories, effectively spanning multiple ontogenetic stages, from mature to senescent and over-mature forms, co-occurring within the same grove. This intrinsic demographic layering gives rise to substantial variation in trunk morphology, crown architecture, and the natural development of cavities and fissures. Together, these features generate a heterogeneous structural template that is ecologically meaningful for cavity-dependent fauna such as *S. anomalus*.

To capture the interaction between *S. anomalus* and centennial olive trees, our study was confined to the island’s High Nature Value farmland (HNVf) olive zones (130.06 km^2^; [55]). These areas were identified by intersecting CORINE Land Cover agricultural data with the Tree Cover Density (TCD) subset of the Copernicus high-resolution layers [79,80], followed by field validation to confirm the presence of terraces, dry-stone walls, and long-term low-intensity management. By restricting sampling to this area, we explicitly targeted landscapes where the ecological role of centennial olive trees could be disentangled from confounding habitats (e.g., pine or oak forests), ensuring that tree-level analyses of den-site selection are grounded in the dominant agroecosystem of the island.

### 2.2. Den Identification and Sampling Design

The identification of den sites was conducted over a four-year period (2022–2025) to ensure temporal replication and minimize interannual bias in detections. Surveys were concentrated during periods of *S. anomalus* peak activity in Lesvos: i.e., spring to early summer (May–June), when dependent young were regularly observed emerging from cavities, providing unambiguous confirmation of reproductive dens (Y.G.Z., pers. obs.; [73]), and early autumn (September), corresponding to a secondary breeding peak (Y.G.Z., pers. obs.). These seasonal windows maximized the probability of detecting active dens while reducing uncertainty in classification. Midwinter and midsummer periods were avoided, as activity levels decline under adverse climatic conditions, increasing the risk of false negatives [81].

Fieldwork was carried out on randomly selected sunny days with moderate temperatures (18–28 °C) to control for weather-related variation in activity. Across the four survey years, all accessible sectors of the HNV olive zone were repeatedly surveyed, ensuring consistent spatial coverage and capturing the full heterogeneity of the traditional grove mosaic. Each survey day was divided into two standardized observation sessions (08:00–12:00 and 16:00–19:00), corresponding to the species’ main bimodal activity peaks [81]. To account for local behavioral variation, we also recorded den activity during midday hours on Lesvos, where squirrels were occasionally active under favorable conditions (Y.G.Z., pers. obs.).

Given the cryptic and arboreal behavior of *S. anomalus*, detection protocols were explicitly designed to minimize human interference and maximize reliability. Observations were conducted from a distance using binoculars, relying on a combination of passive scanning and stationary vantage points to monitor tree crowns and cavities without approaching the focal trees. Surveyors maintained consistent, slow movement and avoided abrupt sounds or gestures, thereby reducing the risk of altering natural activity. These standardized procedures ensured that presence data reflected natural behavior rather than observer-induced disturbance.

**Figure 2:**
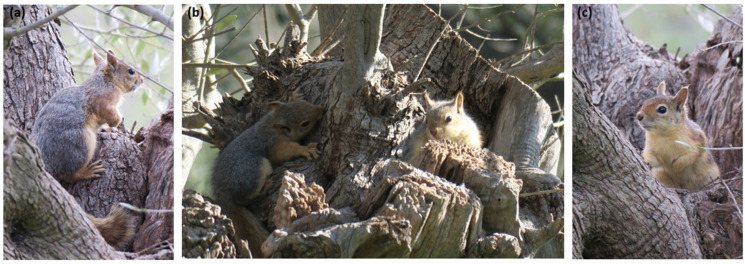
Direct field observation of Persian squirrels (*Sciurus anomalus*) at an olive tree cavity used as a den site: (**a**) adult squirrel resting on the trunk adjacent to the cavity entrance, (**b**) two individuals interacting at the den cavity within the olive tree, (**c**) individual positioned at the cavity entrance, exhibiting alert posture. Observations were conducted with binoculars at distance to minimize disturbance.

Den trees were confirmed exclusively through direct behavioral evidence (Figure 2), including repeated observations of adults entering or exiting cavities, prolonged cavity inspections, or the emergence of dependent young. Ambiguous events (e.g., brief approaches to cavities without entry) were excluded to avoid misclassification. To further minimize false positives, indirect indicators such as feeding remains, tracks, or vocalizations were not used as evidence of denning. Each confirmed den was independently verified by three trained observers (Y.G.Z., E.K., A.C.), while all confirmed den trees were georeferenced using handheld GPS units, with metadata (time, weather, and habitat context) recorded for every detection to ensure traceability.

Through this conservative, non-invasive protocol, a total of 36 den trees were identified (Figure 1). These were distributed across multiple HNV traditional olive groves of Lesvos, ensuring spatial representativeness and capturing variation in topography, microhabitat, and management history.

### 2.3. Plot Establishment and Tree Sampling Design

To assess tree-level traits associated with den-site selection by *S. anomalus*, we implemented a standardized, spatially explicit sampling framework around each of the 36 confirmed den trees. Circular plots with a 50 m radius were delineated using ArcGIS 10.2 (ESRI Inc., Redlands, CA, USA), with each den tree positioned at the center. This spatial scale was selected as an ecologically meaningful approximation of the species’ home range, consistent with movement and foraging behaviors previously documented for *S. anomalus* [41,64,74,82] and for congeneric species such as *S. vulgaris* and *S. carolinensis* [83,84]. The 50 m radius thus represents a biologically relevant domain within which den-site decisions are most likely to be influenced by tree-specific attributes rather than broader landscape-level variables.

Den plots were distributed across multiple olive groves separated by considerable distances (>500 m) (Figure 1), thereby minimizing spatial autocorrelation between sampling units. No grove was sampled more than once for den trees, ensuring plot-level independence in trait measurements and occupancy data.

Within each 50 m plot, seven additional olive trees were selected using a randomized spatial protocol explicitly designed to ensure unbiased sampling. The fixed number of seven trees per plot was chosen to maintain a consistent and ecologically meaningful case-to-availability ratio, capturing local structural variability around each den tree while ensuring sufficient statistical power for trait-based comparisons. All candidate trees within the plot were first mapped, and selection was carried out by overlaying a uniform grid and applying a random number generator to determine which individuals would be included. This procedure minimized spatial autocorrelation, avoided edge effects, and prevented any visual or proximity-based selection bias. To ensure measurement consistency, all 288 trees (36 den trees and 252 non-den trees) were assessed under comparable weather and lighting conditions, thereby reducing potential variability due to external environmental factors.

### 2.4. Structural Traits of Olive Trees

To evaluate the architectural features that may influence den-site selection by *S. anomalus*, we systematically recorded a set of structural traits for all sampled olive trees. All measurements were conducted during the last year of the study, as centennial olive trees exhibit minimal year-to-year variation in structural dimensions due to their extremely slow growth rates [85].

We first quantified the vertical and horizontal dimensions of each olive tree to characterize its overall architecture. Tree height (H, m) was determined with a handheld clinometer, applying trigonometric calculations from a standardized observation distance. Trunk diameter at breast height (DBH, cm) was measured at 1.3 m above ground using a measuring tape, providing a proxy for structural maturity and long-term growth investment. To capture canopy extent, we recorded the major and minor axes of the crown using the vertical sighting method [86] and calculated crown area (CA, m^2^) using established geometric formulas.

Given the species’ dependence on tree hollows for denning, we systematically assessed the abundance of cavities on each olive tree. Only naturally occurring cavities were recorded; pruning wounds or bark loss was excluded to avoid overestimating potential den sites. We therefore adopted a strict definition of cavities, classifying them as naturally formed hollows or vertical fissures with an entrance diameter ≥ 4 cm; this is a size considered sufficient to accommodate adult *S. anomalus* individuals, based on our knowledge of the species’ denning behavior. However, because cavity formation in olive trees is highly age-dependent, raw cavity counts alone may not provide an unbiased index of den suitability. Older trees naturally accumulate structural imperfections over time, irrespective of their ecological function. To account for this, and given the known limitations of ring-counting in centennial olive trees [87], we estimated the age of each tree using a validated allometric equation developed for Mediterranean olives (Age = 2.11 × DBH (cm) + 88.93; R^2^ = 0.80; [88]).

Finally, to control for age-related bias in cavity accumulation and enable meaningful comparisons across trees of varying ontogenetic stages, we computed the Cavity Ratio (CR) for each individual (CR = Number of cavities/Estimated tree age) [55]. This ratio serves as a comparative index of cavity abundance corrected for olive tree age, allowing us to evaluate whether den trees exhibit unusually high cavity formation relative to their developmental stage. In this way, CR reflects not just structural maturity but also potential microhabitat quality; these are both key determinants in den selection by *S. anomalus*.

### 2.5. Physiological and Functional Traits of Olive Trees

To complement the structural assessment, we quantified a set of physiological and functional traits that together provide an integrated profile of tree vitality, canopy productivity, and photosynthetic efficiency.

We first estimated the mean leaf area index (LAI_mean_) of each tree using a SunScan plant canopy analyzer (SunScan System, Delta-T Devices Ltd., Cambridge, UK). For each tree, five replicate measurements were taken at a standardized height of 1.3 m from the ground, distributed at equal distances below the crown to capture canopy heterogeneity. These measurements were averaged to yield LAI_mean_, used here as a proxy for canopy density and overall productivity [89,90]. In addition, we calculated the range of LAI values (LAI_range_) from the same set of measurements, providing an index of crown uniformity; larger LAI_range_ values reflect greater spatial heterogeneity in foliage distribution and are indicative of reduced productivity or crown asymmetry [91].

Because LAI integrates canopy density but not foliage physiology, we further assessed leaf photosynthetic capacity using a hand-held chlorophyll content meter (CCM-200plus, Opti-Sciences, Inc., Hudson, NH, USA). For each tree, we randomly sampled 30 mature leaves from the same crown sectors used for LAI measurements. Each leaf was measured three times, and the average was converted into a Chlorophyll Content Index (CCI) [92]. To allow ecological comparability, we transformed CCI into absolute chlorophyll concentration (g × m^−2^) using the calibration equation of Parry et al. (2014) [93], and then converted values into mg m^−2^ by accounting for the molecular weight of chlorophyll a and b. Multiplying chlorophyll concentration by LAI_mean_ and crown area yielded the estimated total chlorophyll content per tree (TC, g), which reflects whole-tree photosynthetic potential.

To incorporate leaf-level photochemical performance, we measured chlorophyll fluorescence parameters on the same 30 leaves using a portable fluorometer (Pocket PEA, Hansatech Instruments Ltd., King’s Lynn, Norfolk, UK), which were dark-adapted for 20 min before measurement. We recorded the maximum quantum yield of photosystem II (Fv/Fm), widely used as a sensitive index of photoinhibition and stress [94,95]. In parallel, we calculated the performance index (PI), which integrates multiple aspects of photosynthetic efficiency and provides a composite indicator of plant vitality [96]. These parameters complement chlorophyll metrics by quantifying the actual physiological state of the photosynthetic apparatus under field conditions.

Finally, to provide a functional canopy-level measure, we estimated total leaf area (TLA, m^2^) as the product of LAI_mean_ and crown area. This metric captures the total surface area available for light interception, carbon assimilation, and microclimatic buffering, thus serving as an ecologically relevant indicator of both productivity and den-site suitability.

### 2.6. Thermal Traits of Olive Trees

To characterize tree-level microclimatic conditions, we applied infrared thermography (IRT) to both trunks and canopies of all centennial olive trees. Infrared images were acquired using a FLIR T1020 infrared camera (28° optics; FLIR Systems Inc., Wilsonville, OR, USA), following standardized field protocols [14,55,89,91,97].

For each trunk, two images were acquired: Side A, defined in den trees as the orientation containing the active cavity and, in non-den trees, as the side exhibiting the most pronounced structural irregularities (e.g., fissures, bark discontinuities, or superficial perforations). Side B was defined as the opposing, structurally contrasting orientation, providing a standardized reference surface for quantifying thermal symmetry. This approach ensured systematic coverage of both the structurally most relevant and the contrasting sides of the trunk. For each canopy, two additional images were taken at the same orientations (Side A and Side B) at ~45° inclination, providing complementary information on crown-level microclimatic conditions. All images were captured from a fixed distance of 5.0 m and a standardized height of 1.3 m, ensuring consistent spatial resolution and field-of-view across trees.

To reduce atmospheric and radiative noise, the IRT procedure was conducted during early morning hours, when diffuse solar radiation minimized the effects of direct irradiance and within-crown heating heterogeneity [98,99]. All IRT metrics were conducted during late spring and early summer (May–June), a period of peak physiological activity and stable canopy conditions in centennial olive trees, ensuring that thermal traits reflected intrinsic tree properties rather than seasonal or drought-induced variability. Each image was calibrated in the field using concurrent meteorological measurements (ambient air temperature, relative humidity, and solar irradiance) collected beneath each tree canopy with a portable weather station and a solar radiation meter (Amprobe SOLAR-100). Emissivity was fixed at e = 0.95 for trunks [100] and e = 0.98 for canopy [101], values widely validated for woody vegetation.

From each image, histograms of surface temperature were extracted and processed in ArcGIS 10.2 (ESRI Inc., Redlands, CA, USA), while indices were computed to describe the typical central tendency and variability measures. To quantify thermal regimes, we calculated interquartile ranges (IQR) and outer percentile ranges (OPR) for both trunk and canopy, separately for Side A (T_IQR_ Side A, C_IQR_ Side A) and Side B (T_IQR_ Side B, C_IQR_ Side B) of each tree. For a comprehensive overview, we employed interpercentile ranges from each trunk and canopy histogram, collectively forming the outer percentile ranges for both trunk and canopy sides (T_OPR_ Side A, C_OPR_ Side A, T_OPR_ Side B, C_OPR_ Side B). These indices captured the extreme portions of the temperature distribution, thereby reflecting potential anomalies linked to structural features such as cavities, bark fissures, or canopy related abnormalities. Furthermore, to capture potential asymmetry, we also computed side-to-side differences between the trunk and canopy sides (ΔT_IQR_, ΔC_IQR_, ΔT_OPR_, ΔC_OPR_).

Finally, to integrate these multiple metrics into a biologically meaningful framework, we additionally derived a Thermal Buffering Index (TBI) for each tree. This index was calculated as the mean of all trunk and canopy IQR values (T_IQR_ Side A, T_IQR_ Side B, C_IQR_ Side A, C_IQR_ Side B), providing a synthetic measure of microclimatic stability across the entire tree. Lower TBI values indicate stronger buffering capacity and reduced diurnal thermal fluctuations, whereas higher values denote weaker buffering and greater exposure to ambient variability. We retained the mean formulation to (i) give trunk and canopy equal a priori weight at the tree level, (ii) preserve direct ecological interpretability, and (iii) avoid introducing tuning parameters.

### 2.7. Statistical Analyses

We utilized the R statistical environment for comprehensive data analysis (v. 4.4.2, R Core Team, Vienna, Austria). The data were represented as means ± standard deviation, and statistical significance was considered at the 5% level.

We first examined within-individual asymmetries in thermal regimes, aiming to detect directional microclimatic divergence between opposing orientations of each olive tree. Paired *t*-tests were conducted separately for trunk (T_IQR_, T_OPR_) and canopy (C_IQR_, C_OPR_) thermal indices, comparing values between Side A and Side B of the same tree. These tests allowed us to evaluate whether den trees exhibit atypical spatial variability in surface temperature distributions—an indication of possible physiological or structural irregularities [102].

Subsequently, we employed a multiple linear regression model with the Thermal Buffering Index (TBI) as the continuous response variable. Predictors included structural, physiological, and functional traits. Model refinement was conducted through a stepwise removal of the least informative predictors, while monitoring variance inflation factors (VIF) to control for multicollinearity. The final model aimed to identify the key drivers of microclimatic moderation capacity; these are factors that may influence a tree’s value as a thermally buffered refuge for arboreal fauna. In order to illustrate predictor–response relationships we generated effect plots and performed relative importance analysis (RIA) to evaluate the relative explanatory contribution of each retained predictor. Model assumptions (residual distribution and homoscedasticity) were checked to confirm the validity of linear modeling.

Furthermore, to evaluate whether den and non-den trees differ as multivariate profiles, we performed a permutational multivariate analysis of variance (PERMANOVA) using the *adonis2* function in the vegan package [103]. Analyses were based on Bray–Curtis dissimilarities of standardized structural, physiological, and thermal traits, with den presence/absence as the grouping factor. The final multivariate matrix included four structural (H, CA, Age, CR), seven physiological (LAI_mean_, LAI_range_, CCI, TC, Fv/Fm, PI, TLA), and five thermal traits (ΔT_IQR_, ΔT_OPR_, ΔC_IQR_, ΔC_OPR_, TBI), while derived or collinear variables (e.g., DBH, number of cavities) were excluded to avoid redundancy. Homogeneity of multivariate dispersion was assessed with the *betadisper* function to ensure that group separation was not an artifact of unequal within-group variance. To visualize the ordination of trees in multivariate trait space, we conducted a non-metric multidimensional scaling (NMDS) using the same dissimilarity matrix. The significance of individual trait vectors in relation to the ordination axes was assessed with the *envfit* procedure, providing an indication of which variables were most strongly aligned with the observed group differentiation.

Lastly, to identify the structural, physiological, and thermal determinants of den-site selection by *S. anomalus*, we implemented a series of binary logistic regression (BLR) models with den presence (1) vs. absence (0) as the response variable. This modeling structure follows a hierarchical, hypothesis-driven framework appropriate for ecological trait datasets, ensuring interpretability while avoiding overfitting. First, we fitted block-specific models targeting (a) structural traits, (b) physiological and functional traits, and (c) thermal traits. From each block we retained variables that showed consistent and interpretable associations with den presence. These variables were then combined in an exploratory integrative model. To avoid unnecessary complexity and reduce the risk of overfitting, we applied a backward elimination procedure, sequentially removing the least informative variables while monitoring VIF to ensure collinearity control, using a conservative threshold of VIF > 3 for variable exclusion. Model fit was quantified with Nagelkerke’s R^2^ [104], discrimination was assessed using the Area under the Receiver Operating Characteristic Curve (AUC), and the overall goodness-of-fit was evaluated with the Hosmer–Lemeshow statistic [105]. For each final model we used RIA to quantify the standardized contribution of each predictor to the model’s explanatory power.

## 3. Results

### 3.1. Centennial Olive Trees’ Structural and Physiological Traits

The 288 centennial olive trees sampled across High Nature Value olive groves on the island exhibited substantial structural and physiological heterogeneity (Table 1). Mean tree height was 7.0 ± 2.4 m, with a mean DBH of 67.9 ± 32.0 cm supporting crowns of 67.3 ± 34.4 m^2^. Trees contained on average 4.4 ± 2.8 natural cavities, corresponding to an estimated mean age of 232.0 ± 67.7 years. When corrected for ontogenetic stage, cavity formation yielded a mean cavity ratio (CR) of 0.021 ± 0.015, reflecting broad variation in structural microhabitat availability.

Physiological assessments revealed broad variability in canopy productivity and photosynthetic performance among sampled trees. The mean leaf area index (LAI) was 3.00 ± 1.43, while chlorophyll concentration averaged 0.73 ± 0.26 mg m^−2^. At the whole-tree level, chlorophyll content reached 197.4 ± 214.8 g, and total leaf area (TLA) was 225.1 ± 190.1 m^2^, indicating considerable heterogeneity in photosynthetic potential. Photochemical efficiency indices further characterized tree vitality, with mean Fv/Fm of 0.66 ± 0.16 and a performance index (PI) of 5.18 ± 2.58, values consistent with moderate physiological status across the olive trees.

When considered by den status, den trees tended to be taller (10.0 ± 2.4 m) than non-den trees (6.6 ± 2.1 m) and displayed broader crowns (124.7 ± 13.1 m^2^ vs. 59.1 ± 28.1 m^2^) and greater total leaf area (597.3 ± 134.6 m^2^ vs. 171.9 ± 126.8 m^2^). Den trees also exhibited higher chlorophyll concentrations (1.04 ± 0.19 mg m^−2^) and larger whole-tree chlorophyll content (663.8 ± 157.6 g) compared to non-den trees (0.69 ± 0.24 mg m^−2^ and 130.8 ± 116.8 g, respectively), alongside elevated photochemical efficiency (Fv/Fm 0.81 ± 0.03 vs. 0.64 ± 0.16). Conversely, cavity counts were paradoxically lower in den trees (1.9 ± 0.3) than in non-den trees (4.8 ± 2.9), a difference reflected in a substantially lower cavity ratios (CR: 0.008 ± 0.002 vs. 0.022 ± 0.015) (Table 1).

### 3.2. Tree-Level Asymmetries in Trunk and Canopy Thermal Regimes

**Figure 3:**
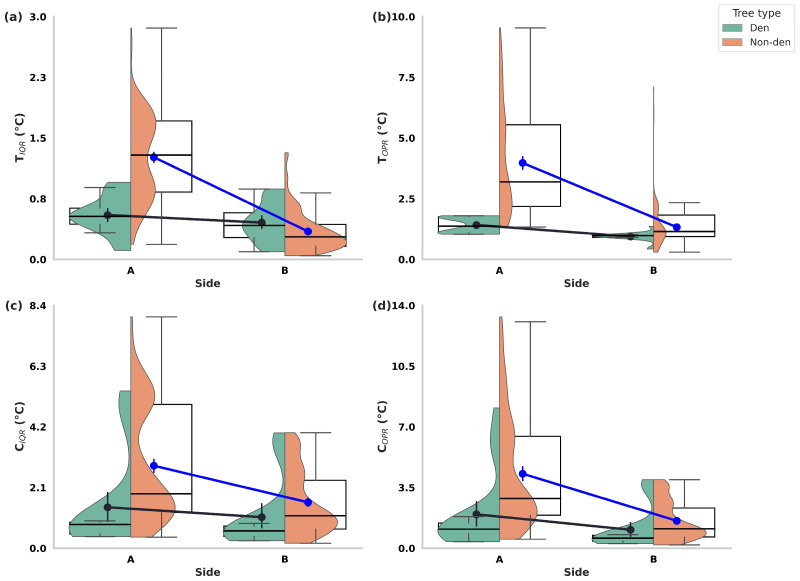
Orientation-related asymmetries in thermal regimes of centennial olive trees (*Olea europaea*) used and not used by Persian squirrels (*Sciurus anomalus*) on Lesvos Island. Each panel depicts trunk and canopy thermal indices of centennial olive trees using hybrid violin–boxplots with overlaid means (±95% CI), comparing Side A and Side B values for den and non-den trees: (**a**) T_IQR_ (Trunk Interquartile Range), (**b**) T_OPR_ (Trunk Outer Percentile Range), (**c**) C_IQR_ (Canopy Interquartile Range), and (**d**) C_OPR_ (Canopy Outer Percentile Range). Violin outlines illustrate the distribution of values, boxplots depict medians and interquartile ranges, and point estimates represent mean values with confidence intervals. Black lines indicate within-tree paired comparisons between Side A and Side B, while blue arrows highlight the directional change in mean thermal values between the two sides. All indices exhibited significantly higher variability on Side A compared to Side B (*p* < 0.001 for all comparisons), indicating consistent orientation-related asymmetries in both trunk and canopy thermal regimes.

IRT revealed consistent orientation-related asymmetries in thermal variability across all sampled olive trees. Trunk indices showed broader distributions on Side A (T_IQR_: 1.17 ± 0.54 °C; T_OPR_: 3.65 ± 2.19 °C) than on Side B (T_IQR_: 0.36 ± 0.26 °C; T_OPR_: 1.28 ± 0.81 °C). Similar patterns were evident in canopy indices, with C_IQR_ averaging 2.67 ± 1.96 °C on Side A versus 1.52 ± 1.11 °C on Side B, and C_OPR_ reaching 4.00 ± 3.16 °C compared to 1.52 ± 1.11 °C on Side B. Paired-sample analyses confirmed that these orientation differences were highly significant (T_IQR_: t(287) = 23.11, *p* < 0.001; T_OPR_: t(287) = 21.46, *p* < 0.001; C_IQR_: t(287) = 14.15, *p* < 0.001; C_OPR_: t(287) = 16.96, *p* < 0.001), indicating a pervasive side-to-side heterogeneity in trunk and canopy thermal regimes (Figure 3).

When examined separately, both den and non-den trees displayed significant asymmetries, though their magnitudes diverged. In den trees, Side A consistently exhibited higher thermal variability than Side B across all indices, whereas non-den trees displayed the same directional pattern but with substantially larger disparities (Table A1).

To further quantify asymmetry, side-to-side difference scores (ΔT_IQR_, ΔC_IQR_, ΔT_OPR_, ΔC_OPR_) were computed. All Δ values were significantly greater than zero, confirming a systematic bias toward higher variability on Side A. Moreover, den trees exhibited consistently smaller Δ indices than non-den trees, indicating that Persian squirrels preferentially occupy trees with reduced side-to-side heterogeneity (Table 2).

### 3.3. Influence of Trees’ Sturctural, Physiological, and Functional Traits on Thermal Buffering Index (TBI)

To identify the determinants of microclimatic buffering capacity, we implemented a multiple linear regression model with backward elimination, retaining only those predictors that maximized explanatory power while controlling for multicollinearity. The final model was highly significant [F(5, 282) = 34.66, *p* < 0.001], with an adjusted R^2^ of 0.370, thereby accounting for more than one-third of the variance in TBI (Table 3). The model incorporated structural (tree height, age) and physiological (LAI_mean_, FV/FM, PI) attributes. Among these, LAI_mean_ emerged as the dominant predictor (β = −0.393, *p* < 0.001), indicating that denser canopies are strongly associated with reduced thermal variability and hence greater buffering stability. Tree height exerted a positive effect (β = 0.265, *p* < 0.001), suggesting that taller trees are characterized by greater thermal instability. Additional but comparatively smaller effects were detected for FV/FM (β = −0.247, *p* < 0.001), PI (β = −0.182, *p* < 0.001), and age (β = −0.137, *p* = 0.008).

Squared part correlations quantified the unique variance in TBI attributable each predictor: LAI_mean_ explained 8.9%, H 4.9%, FV/FM 3.6%, PI 2.8%, and age 1.5%. Together these predictors accounted for ~22% of the variance, while the remaining ~15% was attributable to shared variance among predictors.

Relative importance analysis (RIA), which partitions both unique and shared variance, provided a complementary perspective. LAI_mean_ contributed 31.8% of the explained variance, followed by H (22.7%), FV/FM (18.2%), PI (13.6%), and age (9.1%). While LAI_mean_ remained the dominant predictor across both approaches, the RIA underscored the role of shared variance in amplifying the apparent influence of canopy structure relative to physiological traits.

**Figure 4:**
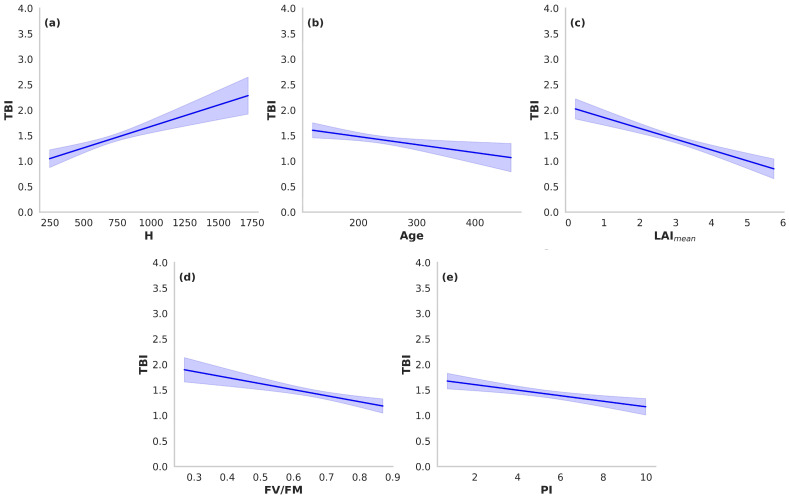
Effect plots illustrating the influence of structural and physiological traits on the Thermal Buffering Index (TBI) of centennial olive trees (*Olea europaea*). Each panel shows the modeled relationship between TBI and a single predictor, holding all other variables constant: (**a**) tree height (H), (**b**) age, (**c**) LAI_mean_, (**d**) FV/FM, and (**e**) performance index (PI). Blue lines represent predicted values, with shaded areas denoting 95% confidence intervals.

To further illustrate these relationships, effect plots were generated for each predictor, displaying modeled responses of TBI while holding all other variables constant (Figure 4). These visualizations reinforce the regression and variance-partitioning results, offering an intuitive depiction of the direction and magnitude of each predictor’s effect on microclimatic buffering capacity in centennial olive trees.

### 3.4. Multivariate Differentiation of Den and Non-Den Olive Trees

A permutational multivariate analysis of variance (PERMANOVA) revealed a strong and highly significant effect of den presence on the overall multivariate composition of centennial olive trees. Den trees differed systematically from non-den trees across the combined set of structural, physiological, and thermal traits, with the model explaining 30.2% of the total variance [F(1, 285) = 123.28, R^2^ = 0.302, *p* = 0.001] (Table A2).

**Figure 5:**
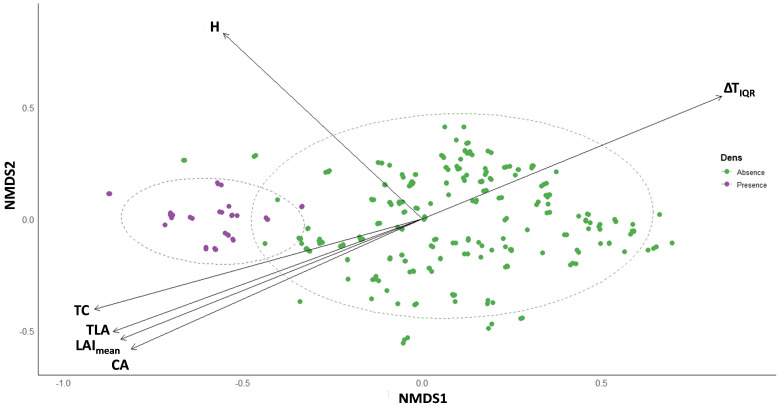
Non-metric multidimensional scaling (NMDS) ordination of centennial olive trees based on Bray–Curtis dissimilarities of standardized structural, physiological, and thermal traits. Trees occupied by *Sciurus anomalus* dens (purple) are clearly separated from unoccupied trees (green), with ellipses representing 95% confidence intervals around group centroids. Overlaid vectors (*p* < 0.05, r^2^ > 0.5) indicate the direction and strength of traits most strongly associated with group differentiation. Taller trees (H) and those with larger crowns (CA), higher mean leaf area index (LAI_mean_), greater total leaf area (TLA), and elevated total chlorophyll (TC) were positively associated with den presence, whereas higher trunk thermal asymmetry (ΔT_IQR_) corresponded to non-den trees, reflecting greater microclimatic instability.

Non-metric multidimensional scaling (NMDS) provided a robust two-dimensional ordination of trait dissimilarities, yielding an excellent representation of the multivariate structure (Figure 5). Trees with dens clustered distinctly from non-den trees, underscoring the strong separation detected by PERMANOVA.

The *envfit* procedure further indicated which traits were most strongly associated with this multivariate differentiation. The attributes with the strongest correlations to ordination axes included H (r^2^ = 0.886, *p* = 0.001), CA (r^2^ = 0.751, *p* = 0.001), TLA (r^2^ = 0.885, *p* = 0.001), and TC (r^2^ = 0.834, *p* = 0.001). Additional contributions were provided by LAI_mean_ (r^2^ = 0.704, *p* = 0.001), CCI (r^2^ = 0.478, *p* = 0.001), Fv/Fm (r^2^ = 0.328, *p* = 0.001), and ΔT_IQR_ (r^2^ = 0.540, *p* = 0.001) (Table A3). The orientation of these vectors within the NMDS ordination illustrates the direction and strength of their association with the observed group separation (Figure 5). Tree height (H) projected upward along the NMDS2 axis, indicating that den trees tended to be taller than non-den counterparts. CA, TLA, LAI_mean_, and TC were all oriented toward the negative NMDS1 axis, suggesting that den trees were generally characterized by broader canopies, larger photosynthetic surface area, and greater canopy-level photosynthetic capacity. By contrast, the ΔT_IQR_ aligned strongly along the positive NMDS1 axis, showing that trees with pronounced side-to-side thermal heterogeneity were positioned away from den trees and thus more typical of non-den olive trees.

### 3.5. Determinants of Den-Site Selection by Sciurus Anomalus

Multivariate analyses (see Section 3.4) confirmed that den and non-den trees occupy relatively distinct positions in multidimensional trait space, with group separation strongly aligned with structural, physiological, and thermal traits. To identify which specific attributes most strongly explained this differentiation, we fitted block-specific and integrative BLR models (summary in Table A4).

The BLR model assessing the structural traits of centennial olive trees as predictors of den-site selection by *S. anomalus* was statistically significant [χ^2^(2, N = 288) = 177.65, *p* < 0.001; Table 4a], confirming that crown area (CA) and cavity ratio (CR) exerted strong and opposing effects on the probability of den occupation. The model demonstrated high explanatory power, accounting for 87.0% of the variance in den presence (Nagelkerke R^2^ = 0.870). Validation analyses reinforced the robustness of the model. The ROC curve yielded an AUC of 0.994 ± 0.003 (95% CI: 0.987–1.000, *p* < 0.001), indicating near-perfect discriminatory capacity in distinguishing den from non-den trees. Classification accuracy was high, with an overall success rate of 96.5% based on an ROC-optimized cutoff (0.227), correctly classifying 96.4% of non-den trees and 97.2% of den trees. The Hosmer–Lemeshow test was non-significant (χ^2^(8) = 0.283, *p* = 0.91), confirming excellent model calibration and no detectable deviation between observed and expected frequencies. Among the structural attributes, CA exerted a strong positive influence on den-site use, with each additional square meter of canopy increasing the odds of occupation by ~19%. By contrast, CR had a marked negative effect, indicating that trees with accelerated rates of cavity formation were almost never selected. Relative Importance Analysis (RIA) revealed that crown area accounted for 54.9% of the model’s explanatory power, while cavity ratio contributed 45.0%.

The model incorporating physiological and functional attributes was significant [χ^2^(2, N = 288) = 183.61, *p* < 0.001; Table 4b], explaining 89.1% of the variance in den presence (Nagelkerke R^2^ = 0.891). Model calibration was acceptable (Hosmer–Lemeshow χ^2^(8) = 3.07, *p* = 0.93), discrimination was high (AUC = 0.994 ± 0.004, 95% CI: 0.987–1.000, *p* < 0.001), and the ROC-optimized cutoff (0.371) yielded 98.3% overall accuracy (98.8% of non-den and 94.4% of den trees correctly classified). Backward elimination retained total chlorophyll (TC) and total leaf area (TLA) as positive predictors of den occupation (Table 4b), while Relative Importance Analysis (RIA), computed as each predictor’s Wald χ^2^ divided by the sum of Wald χ^2^ in the final model, indicated TC = 66.0% and TLA = 34.0% of the explained contribution, suggesting that canopy-level photosynthetic capital plays a somewhat larger role than canopy surface area in differentiating den from non-den trees within this trait set.

The model based on thermal asymmetry indices was also highly significant [χ^2^(2, N = 288) = 204.47, *p* < 0.001; Table 4c], explaining 96.0% of the variance in den presence (Nagelkerke R^2^ = 0.960). Model fit and calibration were excellent (Hosmer–Lemeshow χ^2^(8) = 0.129, *p* = 0.91), and classification accuracy reached 99.0% at the ROC-optimized cutoff (0.535), correctly classifying 99.2% of non-den and 97.2% of den trees. The ROC curve confirmed near-perfect discrimination (AUC = 0.999 ± 0.001, 95% CI: 0.997–1.000, *p* < 0.001). Both retained predictors (ΔT_IQR_ and ΔT_OPR_) exerted strong negative effects (ΔT_IQR_: B = −30.19, SE = 13.50, Wald = 5.00, *p* = 0.025, Exp(B) ≈ 0; ΔT_OPR_: B = −48.25, SE = 20.55, Wald = 5.51, *p* = 0.019, Exp(B) ≈ 0). These results indicate that trees with pronounced orientation-related asymmetries in trunk and canopy thermal variability were systematically avoided as den sites. Relative Importance Analysis (RIA), based on the standardized Wald χ^2^ contributions, attributed 47.6% of the explanatory weight to ΔT_IQR_ and 52.4% to ΔT_OPR_, underscoring that both trunk and canopy thermal instability equally constrained den-site selection.

The exploratory integrative model initially incorporated candidate predictors retained from the structural (CA, CR), physiological/functional (TC, TLA), and thermal (ΔT_IQR_, ΔT_OPR_) models. Despite the broader set of variables considered, backward elimination procedures retained only crown area (CA) and trunk thermal asymmetry (ΔT_IQR_) in the final model [χ^2^(2, N = 288) = 172.77, *p* < 0.001; Table 4d]. The model explained 85.2% of the variance in den presence (Nagelkerke R^2^ = 0.852), with excellent calibration (Hosmer–Lemeshow χ^2^(8) = 0.382, *p* > 0.05) and strong discriminatory power (AUC = 0.990 ± 0.004, 95% CI: 0.982–0.998, *p* < 0.001). At the ROC-optimized cutoff (0.181), overall classification accuracy was 94.4%, correctly identifying 94.0% of non-den and 97.2% of den trees.

Within this integrative framework, crown area maintained a strong positive association with den occupation, whereas ΔT_IQR_ exerted a significant negative influence. Relative Importance Analysis (RIA) attributed 51.5% of the explained contribution to CA and 48.5% to ΔT_IQR_, emphasizing that both expansive crowns and thermal stability acted as equally decisive filters for den-site selection. The exclusion of cavity ratio, chlorophyll-based indices, and canopy-level leaf area in the final step indicates that their explanatory capacity was largely redundant when structural canopy size and trunk thermal heterogeneity was simultaneously accounted for.

## 4. Discussion

In this study, we present the first empirically grounded assessment of den-site selection in the Persian squirrel (*Sciurus anomalus*) within a traditional, insular Mediterranean landscape, using a four-year dataset that integrates structural, physiological, and thermal measurements at the scale of individual trees. Rather than inferring habitat preference from coarse proxies such as vegetation types [41,62,74,81], or canopy cover [106], our work captures the mechanistic coupling between tree state and microclimatic stability; these are the two axes that govern energetic efficiency and predator avoidance in arboreal mammals [107,108], thereby providing the first quantitative and replicable model of tree-level thermal buffering and architectural complexity in den-site selection. This integrative framework not only refines our understanding of the functional ecology and habitat determinants of *S. anomalus* within one of the habitats it uses on the island, but also establishes a quantitative foundation for conservation planning in insular and fragmented Mediterranean landscapes, where restricted dispersal, demographic isolation, and climatic instability converge to elevate extinction risk [109,110]. Equally significant, it constitutes the first empirical demonstration that centennial olive trees act as biophysical refugia, providing structural, physiological, and thermal conditions essential for the persistence of arboreal mammals at the climatic and biogeographic margins of their range. On Lesvos, where *S. anomalus* persists as a small, peripheral population of roughly 500–3000 individuals [111], viability is governed not only by the spatial extent of suitable habitat [41] but also by the functional integrity of individual trees capable of providing enduring structural and microclimatic refuge. Such niche filtering is characteristic of range-edge and insular populations, where a variety of pressures impose strong selection on habitat use and behavioral plasticity [112,113,114,115,116,117,118].

On Lesvos, these interacting pressures are further intensified by land use transitions that have progressively reshaped the traditional olive landscape, one of the core, and arguably the most important, habitat of *S. anomalus* on the island. Until the 1990s, olive cultivation followed extensive, low-intensity practices in which, farmers shaped trees to attain considerable height and broad crown area [55]. Over the past three decades, however, the combined effects of agricultural abandonment [16], socio-economic decline [119], and the partial mechanization of harvesting practices have transformed this dynamic system. Many groves are now only sporadically managed or entirely abandoned (Y.G.Z., pers. obs.), while others undergo occasional deep pruning not only to facilitate olive collection but also to obtain firewood for household or commercial use, thereby diminishing the trees’ structural and functional complexity [24,120].

Beyond these direct disturbances, the physiological and architectural senescence of long-neglected trees further compromises their ecological function. Chronic wood decay and phloem transport depression induce a progressive shift of photosynthetic tissue toward the outer crown [121], leading to an open, light-dominated canopy architecture that replaces the dense, thermally stable canopies once characteristic of mature olive trees. This transition diverts resources toward non-productive branches, diminishes reproductive vigor, and collapses the internal canopy complexity which is critical for shelter and thermal regulation [120]. Within this altered structural context, our trait-based analyses reveal a clear ecological filter in den-site selection: *S. anomalus* consistently occupied centennial olive trees exhibiting better structural and physiological performance within the groves (Table 1).

The apparent paradox that den trees contained fewer cavities than non-den trees is consistent with selection for quality over quantity; although we did not quantify predator-avoidance attributes (e.g., cavity height or entrance geometry), the pattern clearly indicates that *S. anomalus* favors structurally secure cavities within physiologically robust trees. For *S. anomalus*, a usable den is not “any cavity”, but one that couples safe geometry (adequate height, narrow entrance, sufficient depth, and appropriate orientation) with a physiologically stable canopy (dense, live crown and high photosynthetic performance) that buffers microclimate and sustains concealment [69,73]. In centennial olive trees, gnarled trunks frequently develop multiple superficial fissures and perforations which, when numerous, can vent the cavity, increase light and airflow, and create additional access points for predators. These features reduce insulation and security, properties repeatedly linked to lower use and success among cavity-nesting species [122,123,124]. Accordingly, *S. anomalus* appears to prioritize the microclimatic and anti-predator properties of a small number of high-quality cavities within robust trees over the sheer availability of many, thermally “leaky”, or overly exposed openings. This pattern, fully in line with adaptive nest/den-site selection, focused on thermal buffering, energetic economy, and predator avoidance [69,123,125].

Such a selective pattern naturally raises the question of why certain trees sustain the stable microclimatic conditions that make them repeatedly favored by *S. anomalus*. To address this, we moved beyond structural attributes and examined whether the physiological state of centennial olive trees (quantified through IRT) could explain the subtle yet ecologically decisive differences between den and non-den trees. Infrared thermography, by capturing the fine-scale thermal dynamics of living tissues, allows a direct assessment of a tree’s internal functional coherence, revealing how heat and water are distributed and regulated within the trunk and canopy [14,55,89,91,97,102,126].

In this regard, the level of thermal symmetry or asymmetry that exists between trunk and canopy sides can be both a physical descriptor indicating the vigor of hydraulic performance, compartmentalization efficiency and overall vitality in trees, as well as a diagnostic signal of water transport rates through xylem trunks. The orientation-dependent thermal asymmetries observed in trunks and canopies demonstrate a physiological dissimilarity among centennial olive trees regardless of the irradiance effects. Since early-morning light, under which all the IRT images were taken, has very little direct solar heating, the interquartile ranges (T_IQR_, C_IQR_) and outer percentile ranges (T_OPR_, C_OPR_) observed on Side A cannot be attributable to illumination bias. Instead, they reflect intrinsic asymmetries in heat conduction, water transport, and tissue integrity which are core properties of the olive tree’s hydraulic system. In centennial olive trees, localized xylem dysfunction, bark discontinuities, and internal cavities alter the internal flow of water and heat, producing uneven surface temperatures even under uniform ambient conditions [14,48,127]. Thus, elevated T_IQR_ and T_OPR_ values do not capture random noise but rather the physiological footprint of internal irregularities, incomplete compartmentalization, or decay boundaries [43,128,129].

When considered separately for den and non-den trees, the pattern becomes even more revealing. Den trees consistently exhibited smaller side-to-side differences (ΔT_IQR_, ΔT_OPR_, ΔC_IQR_, ΔC_OPR_), indicating a higher level of thermal symmetry across trunk and canopy orientations. This symmetry denotes an efficiently regulated thermal regime, characteristic of trees maintaining strong hydraulic continuity, high transpiration activity, and intact structural compartmentalization [52,102]. In contrast, the large Δ values of non-den trees point to localized hydraulic stress, partial tissue decay, or disrupted water pathways that compromise their thermohydraulic stability. From a diagnostic perspective, the IQR-based indices quantify general variability within the trunk and canopy temperature range, while the OPR-based indices reveal the amplitude of extreme deviations; these metrics are already established as highly sensitive indicators of the tree’s functional integrity [14].

Ecologically, the lower asymmetry observed in den trees implies that *S. anomalus* preferentially selects trees that maintain a physiologically stable microclimate throughout their structure. Such trees offer thermal buffering for cavity interiors, reducing diurnal fluctuations in temperature and humidity. Therefore, the reduced Δ indices in den trees signify ecological suitability: squirrels exploit trees that function as living thermal stabilizers, whose internal equilibrium directly translates into nest-level comfort and safety. Collectively, the coordinated behavior of T_IQR_, T_OPR_, C_IQR_, and C_OPR_ demonstrates that den trees are thermally coherent, hydraulically functional, and structurally intact individuals within the grove system. These findings position IRT-derived thermal indices as a powerful non-invasive diagnostic for both tree health [55,91,102,130,131] and tree-level den-site suitability in traditional agroforestry landscapes.

To integrate the multiple thermal metrics into a single framework, the Thermal Buffering Index (TBI) was developed as an integrated descriptor of whole-tree microclimatic coherence, and revealed that the ability of centennial olive trees to stabilize internal microclimates is driven primarily by canopy functionality rather than by mere size or age. Among all predictors, LAI_mean_ emerged as the dominant factor governing thermal moderation; trees with denser canopies achieve stronger radiative shielding and maintain sustained transpirational cooling, thereby minimizing temperature fluctuations between trunk and crown [106,132,133]. The concurrent influence of photochemical efficiency (FV/FM) and performance index (PI) confirmed that physiological vigor reinforces this structural buffering by sustaining stomatal conductance and hydraulic continuity under fluctuating environmental conditions, consistent with the broader literature on tree physiological function [48,95,134,135]. These functional and structural traits operate synergistically: dense foliage provides the structural platform for evapotranspirative cooling, while physiological vigor ensures that water fluxes remain continuous, jointly producing a self-regulated thermal envelope around the tree. Conversely, taller trees exhibited weaker buffering (higher TBI), reflecting increased convective and radiative exposure that limits the canopy’s ability to maintain uniform temperature profiles [136,137]. The modest negative influence of age likely mirrors senescence-related deterioration in xylem continuity and compartmentalization capacity [120,121]. While the model explained 37% of the variance, this proportion is substantial for an index as integrative and physiologically complex as thermal buffering, indicating that the measured variables capture the core determinants of microclimatic stability.

Despite having established the proximate mechanisms governing the thermal buffering of individual trees, den-site selection by *S. anomalus* ultimately reflects the integrated expression of structural, physiological, and thermal attributes acting together rather than in isolation. To examine how these dimensions converge into a coherent ecological signal, we expanded the analysis to a multivariate perspective to determine whether den trees occupy a distinct region within the multidimensional trait space defined by structural, physiological, and thermal metrics. The result of the PERMANOVA demonstrated that den occupancy is not random but reflects a coherent multivariate signature integrating structural, physiological, and thermal dimensions. The NMDS ordination provides a visualization of this pattern: den trees cluster within a specific region of multivariate space defined by high tree height, large crown and total leaf area, elevated chlorophyll content, and low trunk thermal asymmetry. These axes collectively describe a pattern of structural dominance and physiological vitality coupled with thermal coherence; these conditions that together define the “functional optimum” for den occupancy. The orientation of ΔT_IQR_ away from den trees highlights that thermal heterogeneity acts as a repelling factor, consistent with the principle that internal thermal instability reflects compromised hydraulic or compartmental integrity [14,91]. Thus, the multivariate differentiation aligns precisely with the trait-level and TBI-based interpretations. Functionally, this separation illustrates that *S. anomalus* selects den sites not merely based on geometric or cavity attributes but through the integrated perception of tree vitality and microclimatic reliability, a pattern consistent with broader evidence that cavity-using vertebrates balance structural security and thermal stability when selecting refugia [138], thereby reinforcing the view that centennial olive trees with such traits operate as physiologically active refugia within traditional Mediterranean agroecosystems.

While the multivariate analyses delineated a clear ecological separation between den and non-den trees within the multidimensional trait space, they did not reveal the relative weight or direction of the underlying drivers. To disentangle these contributions and identify which attributes exert the strongest influence on den occupation, we employed block-specific and integrative binary logistic regression models. Among structural traits, crown area emerged as the single most powerful positive determinant, confirming that expansive canopies substantially enhance the probability of den occupancy. Expansive crowns provide both the spatial architecture for concealment and locomotion and the aerodynamic and radiative balance required to sustain internal microclimatic constancy; these traits repeatedly associated with reproductive success in arboreal mammals [139,140]. The avoidance of trees with excessive cavities reinforces that *S. anomalus* follows a functionality gradient rather than a mere resource gradient. The physiological traits, particularly total chlorophyll and total leaf area, further captured the canopy’s photosynthetic capacity and hydraulic resilience, core axes of the plant economic spectrum that underpin the ability for evaporative cooling and energy balance under Mediterranean climate [141,142]. More than that, the thermal attributes provided the mechanistic bridge linking structure and physiology. Both trunk and canopy asymmetry indices (ΔT_IQR_, ΔT_OPR_) exerted strong negative effects on den probability, revealing that even subtle disruptions in internal thermohydraulic coherence render a tree unsuitable. Elevated asymmetry reflects impaired water flux and compartmental decay [14,127], conditions that destabilize tree health. The integrative model distilled this complexity into two decisive axes, the crown area and the trunk thermal asymmetry, demonstrating that *S. anomalus* selects trees that behave as self-regulated biophysical systems whose structure and function are optimized for both persistence and refuge provision.

From a conservation perspective, this hierarchy delineates actionable priorities. The same traits that predict den occupancy (large crowns, dense foliage, and thermally coherent trunks) also define the resilience of centennial olive trees to projected climatic scenarios [143,144] at the landscape scale. Traditional pruning regimes that preserve crown volume, avoidance of over-mechanized harvests, and the monitoring of thermal asymmetry as a health index could serve as practical, evidence-based conservation measures.

Conceptually, these findings advance a paradigm shift from the conservation of habitat forms to the conservation of habitat functions. Den trees emerge not as static structural features but as living, self-regulating systems whose physiological integrity determines their capacity to act as refugia. This functional perspective aligns with contemporary conservation theory emphasizing process-based rather than pattern-based approaches to ecosystem resilience [39]. Within this framework, the Persian squirrel acts as a functional indicator species, its den-site choices empirically revealing the biophysical parameters that sustain arboreal life within traditional olive landscapes. Thus, the “guide to the olive galaxy” is not just a metaphor; it encapsulates an empirically defined framework for identifying and prioritizing trees that maintain the ecological functionality that is essential for the persistence of biodiversity within traditional Mediterranean landscapes.

## 5. Conclusions

In this study, we provided the first process-based, tree-level assessment of den-site selection by *Sciurus anomalus* in traditional insular Mediterranean olive groves. Our results indicate that squirrels select living trees that function as self-regulating biophysical units, in which structure and physiology jointly sustain stable, protective microclimates. Practically, these findings translate into clear, testable conservation actions within High Nature Value olive landscapes. Management that preserves crown volume, avoids deep or over-mechanized pruning, and maintains canopy density will sustain the structural and physiological conditions linked to den occupancy. Non-invasive thermal metrics (ΔT_IQR_, ΔT_OPR_, and TBI) can serve as operational indicators for prioritizing, monitoring, and auditing individual trees, enabling rapid triage of high-value “den-ready” trees at grove and landscape scales. While our inference is robust for an insular, traditional system, it remains observational and geographically bounded. Future research should therefore validate the proposed indicators in other tree species used by *S. anomalus* for denning and temporary refuge from predators, such as chestnut (*Castanea sativa*) and oak (*Quercus* spp.), across different habitat types. Such comparative analyses would clarify whether the functional traits identified here represent a species-specific preference linked to olive grove architecture or a broader adaptive strategy for microclimatic and anti-predator optimization. By shifting attention from habitat form to habitat function, our framework provides a scalable and replicable basis for integrating tree-physiological performance into wildlife management and for safeguarding the microclimatic refugia that underpin the persistence of *S. anomalus* and co-occurring arboreal fauna in Mediterranean agroecosystems. More broadly, the demonstration that individual trees operate as functional biophysical units illustrates a principle of far wider relevance. Conservation strategies across habitats and taxa can benefit from this functional lens, prioritizing not only where habitat exists, but whether it actively performs the buffering, protective, and resilience-enhancing roles required by wildlife. Framing habitat as a dynamic physiological resource rather than a static structural category thus offers a generalizable pathway for strengthening conservation planning under accelerating climatic and land use change.

## Figures and Tables

**Table 1:** Descriptive statistics (mean ± SD) of structural and physiological traits of centennial olive trees used (den) and not used (non-den) by *Sciurus anomalus* on Lesvos Island.

Trait	All Trees (n = 288)	Den Trees (n = 36)	Non-Den Trees (n = 252)	Units
Height (H)	7.03 ± 2.44	10.02 ± 2.39	6.60 ± 2.13	m
DBH	67.9 ± 32.0	78.7 ± 23.7	66.3 ± 32.7	cm
Crown area (CA)	67.3 ± 34.4	124.7 ± 13.1	59.1 ± 28.1	m^2^
Cavities	4.41 ± 2.84	1.89 ± 0.32	4.77 ± 2.85	count
Age	232.0 ± 67.7	255.5 ± 50.3	228.7 ± 69.3	years
CR	0.021 ± 0.015	0.008 ± 0.002	0.022 ± 0.015	cavities/age
LAI_mean_	3.00 ± 1.43	5.10 ± 0.36	2.70 ± 1.27	-
LAIr_ange_	2.42 ± 1.09	1.81 ± 0.86	2.51 ± 1.09	-
CCI	0.73 ± 0.26	1.04 ± 0.19	0.69 ± 0.24	mg m^−2^
TC	197.4 ± 214.8	663.8 ± 157.6	130.8 ± 116.8	g
Fv/Fm	0.66 ± 0.16	0.81 ± 0.03	0.64 ± 0.16	-
PI	5.18 ± 2.58	7.89 ± 1.80	4.79 ± 2.44	-
TLA	225.1 ± 190.1	597.3 ± 134.6	171.9 ± 126.8	m^2^

**Table 2:** Side-to-side asymmetry indices (Δ = Side A − Side B) for trunk and canopy thermal variables in centennial olive trees used and not used by Persian squirrels on Lesvos Island. Values are presented as mean ± SE.

Index	All Trees Δ (vs. 0)	t(df), *p*	Den Trees (M ± SE)	Non-Den Trees (M ± SE)	t(df), *p* (Den vs. Non-den)
ΔT_IQR_	0.81 ± 0.03	25.7(287), <0.001	0.09 ± 0.01	0.92 ± 0.04	−21.8(285), <0.001
ΔT_OPR_	2.33 ± 0.10	23.5(287), <0.001	0.47 ± 0.05	2.64 ± 0.12	−17.1(285), <0.001
ΔC_IQR_	1.12 ± 0.08	13.6(287), <0.001	0.34 ± 0.08	1.26 ± 0.09	−7.6(285), <0.001
ΔC_OPR_	2.47 ± 0.12	20.7(287), <0.001	0.89 ± 0.18	2.71 ± 0.16	−7.7(285), <0.001

**Table 3:** Final multiple linear regression model predicting the Thermal Buffering Index (TBI) of centennial olive trees. The slope of the predictor variable for the response variable (B), the standard error for the slope (SE B), the standardized beta (β), the *t*-test statistic (t), the probability value (*p*), the regression-adjusted coefficient for the regression model (R^2^), and the predictive capability of the model (F) are presented.

Response Variable	Predictor Variables	B	SE B	β	t	*p*-Value	R^2^_adj._	F
TBI	(constant)	2.913	0.200		14.584	0.001	0.37	34.658
	H	0.001	0.000	0.265	4.746	0.001		
	Age	−0.002	0.001	−0.137	−2.656	0.008		
	LAI_mean_	−0.212	0.033	−0.393	−6.358	0.001		
	FV/FM	−1.187	0.295	−0.247	−4.027	0.001		
	PI	−0.054	0.015	−0.182	−3.563	0.001		

**Table 4:** Logistic regression models predicting den-site selection of *Sciurus anomalus*. Β = logistic coefficient; S.E. = standard error of estimate; Wald = Wald chi-square; df = 1 (in all models); *p*-value = significance.

**(a) Structural Traits**				
**Predictor**	**Β**	**S.E.**	**Wald’s χ^2^**	***p*-Value**
Crown area (CA)	0.175	0.042	17.03	<0.001
Cavity ratio (CR)	−454.27	121.64	13.95	<0.001
Constant	−15.37	4.02	14.62	<0.001
**(b) Physiological and Functional Traits**				
**Predictor**	**Β**	**S.E.**	**Wald’s χ^2^**	***p*-Value**
Total chlorophyll (TC)	0.016	0.004	14.90	<0.001
Total leaf area (TLA)	0.013	0.005	7.68	0.006
Constant	−13.67	3.46	15.65	<0.001
**(c) Thermal Traits**				
**Predictor**	**Β**	**S.E.**	**Wald’s χ^2^**	***p*-Value**
ΔT_IQR_	−30.19	13.50	5.00	0.025
ΔT_OPR_	−48.25	20.55	5.51	0.019
Constant	42.13	17.56	5.76	0.016
**(d) Integrative Model**				
**Predictor**	**Β**	**S.E.**	**Wald’s χ^2^**	***p*-Value**
Crown area (CA)	0.174	0.044	15.49	<0.001
ΔT_IQR_	−5.96	1.56	14.58	<0.001
Constant	−18.69	4.83	14.95	<0.001

## Data Availability

The datasets generated and analyzed during the current study are available from the corresponding author upon reasonable request. The data are part of a broader, ongoing research initiative on the species distribution and ecology, and are therefore not publicly released at this stage to ensure data integrity and consistency across related analyses.

## Abbreviations

The following abbreviations are used in this manuscript:

HNVf	High Nature Value farmlands
H	Tree height
DBH	Diameter at breast height
CA	Crown area
CR	Cavity ratio (number of cavities/estimated tree age)
LAI	Leaf area index
LAI_mean_	Mean leaf area index (average LAI per tree)
LAI_range_	Range of leaf area index values within a tree crown
CCI	Chlorophyll Content Index
TC	Total chlorophyll content (per tree)
Fv/Fm	Maximum quantum yield of photosystem II
PI	Performance index (composite chlorophyll fluorescence index)
TLA	Total leaf area
IRT	Infrared thermography
IQR	Interquartile range (of surface temperature)
OPR	Outer percentile range (of surface temperature)
T_IQR_	Trunk interquartile range
T_OPR_	Trunk outer percentile range
C_IQR_	Canopy interquartile range
C_OPR_	Canopy outer percentile range
ΔT_IQR_	Side-to-side difference in trunk IQR (Side A-Side B)
ΔT_OPR_	Side-to-side difference in trunk OPR (Side A-Side B)
ΔC_IQR_	Side-to-side difference in canopy IQR (Side A-Side B)
ΔC_OPR_	Side-to-side difference in canopy OPR (Side A-Side B)
TBI	Thermal Buffering Index (mean of trunk and canopy IQR values)

## Appendix A

**Table A1:** Paired *t*-tests comparing thermal variability between tree orientations (Side A vs. Side B) for den and non-den centennial olive trees. Values represent mean ± standard deviation for each thermal index, accompanied by t statistic (t, degrees of freedom) and associated *p*-values. All indices (T_IQR_, T_OPR_, C_IQR_, C_OPR_) showed significantly greater variability on Side A, with non-den trees exhibiting substantially higher asymmetry than den trees.

Group	Index	Side A (Mean ± SD)	Side B (Mean ± SD)	t (df)	*p*-Value
Den trees	T_IQR_	0.55 ± 0.24	0.46 ± 0.24	t(35) = 7.49	<0.001
	T_OPR_	1.41 ± 0.31	0.94 ± 0.18	t(35) = 9.38	<0.001
	C_IQR_	1.41 ± 1.65	1.07 ± 1.20	t(35) = 4.19	<0.001
	C_OPR_	1.95 ± 2.21	1.06 ± 1.21	t(35) = 5.09	<0.001
Non-den trees	T_IQR_	1.26 ± 0.51	0.35 ± 0.25	t(251) = 25.64	<0.001
	T_OPR_	3.97 ± 2.15	1.33 ± 0.86	t(251) = 22.69	<0.001
	C_IQR_	2.85 ± 1.94	1.58 ± 1.09	t(251) = 14.09	<0.001
	C_OPR_	4.29 ± 3.17	1.58 ± 1.08	t(251) = 16.89	<0.001

**Table A2:** PERMANOVA results (Bray–Curtis dissimilarities) comparing the multivariate structural, physiological, and thermal trait composition of den and non-den centennial olive trees.

Source	Df	Sum of Squares	Mean Squares	F-Value	R^2^	*p*-Value
Den presence	1	3.8264	3.8264	123.28	0.30194	0.001
Residual	285	8.8463	0.03104		0.69806	
Total	286	12.6727			1	

**Table A3:** Results of the envfit analysis, presenting the strength (r^2^) and direction of structural, physiological, and thermal trait vectors fitted to the NMDS ordination, indicating which variables most strongly contribute to the multivariate separation of den and non-den centennial olive trees.

Variable	NMDS1	NMDS2	r^2^	*p*-Value
H	−0.555	0.832	0.886	0.001
CA	−0.813	−0.582	0.751	0.001
Age	−0.569	0.822	0.221	0.001
CR	0.993	0.113	0.193	0.001
LAI_mean_	−0.842	−0.540	0.704	0.001
LAI_range_	−0.081	−0.997	0.039	0.003
CCI	−0.833	−0.554	0.477	0.001
TC	−0.914	−0.405	0.834	0.001
Fv/Fm	−0.862	−0.507	0.327	0.001
PI	−0.998	−0.063	0.142	0.001
TLA	−0.863	−0.506	0.885	0.001
ΔT_IQR_	0.835	0.550	0.540	0.001
ΔT_OPR_	0.803	0.596	0.127	0.001
ΔC_IQR_	0.619	0.785	0.222	0.001
ΔC_OPR_	0.454	0.891	0.223	0.001
TBI	0.623	0.782	0.213	0.001

**Table A4:** Summary of binary logistic regression (BLR) models predicting den-site selection by *Sciurus anomalus*. The table consolidates key predictors, effect directions, model performance metrics, and classification accuracy values across all structural, physiological/functional, thermal, and integrative models.

Model	Retained Predictors	Effect Direction	Nagelkerke R^2^	AUC	Overall Accuracy
Structural traits	CA, CR	CA (+), CR (−)	0.87	0.994	96.50%
Physiological traits	TC, TLA	TC (+), TLA (+)	0.89	0.994	98.30%
Thermal traits	ΔT_IQR_, ΔT_OPR_	ΔT_IQR_ (−), ΔT_OPR_ (−)	0.96	0.999	99.00%
Integrative model	CA, ΔT_IQR_	CA (+), ΔTI_QR_ (−)	0.85	0.990	94.40%
